# Thymic Crosstalk Coordinates Medulla Organization and T-Cell Tolerance Induction

**DOI:** 10.3389/fimmu.2015.00365

**Published:** 2015-07-20

**Authors:** Noëlla Lopes, Arnauld Sergé, Pierre Ferrier, Magali Irla

**Affiliations:** ^1^Centre d’Immunologie de Marseille-Luminy, INSERM, U1104, CNRS UMR7280, Aix-Marseille Université UM2, Marseille, France; ^2^Centre de Recherche en Cancérologie de Marseille, Institut Paoli-Calmettes, INSERM U1068, CNRS UMR7258, Aix-Marseille Université UM105, Marseille, France

**Keywords:** autoimmune regulator, dendritic cells, medulla, medullary thymic epithelial cells, natural regulatory T cells, negative selection, T-cell tolerance, thymic crosstalk

## Abstract

The thymus ensures the generation of a functional and highly diverse T-cell repertoire. The thymic medulla, which is mainly composed of medullary thymic epithelial cells (mTECs) and dendritic cells (DCs), provides a specialized microenvironment dedicated to the establishment of T-cell tolerance. mTECs play a privileged role in this pivotal process by their unique capacity to express a broad range of peripheral self-antigens that are presented to developing T cells. Reciprocally, developing T cells control mTEC differentiation and organization. These bidirectional interactions are commonly referred to as thymic crosstalk. This review focuses on the relative contributions of mTEC and DC subsets to the deletion of autoreactive T cells and the generation of natural regulatory T cells. We also summarize current knowledge regarding how hematopoietic cells conversely control the composition and complex three-dimensional organization of the thymic medulla.

## Introduction

**Figure 1:**
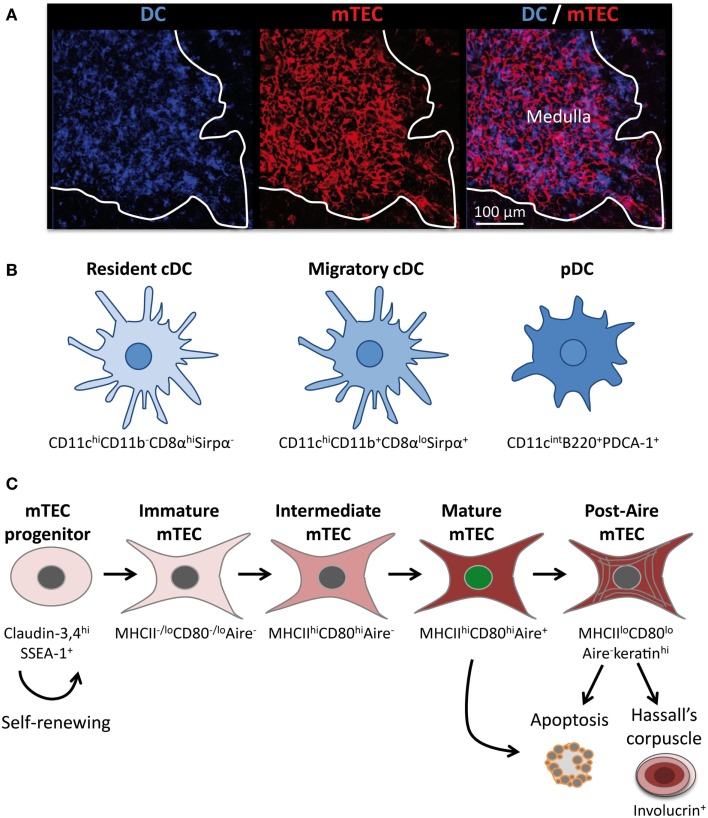
**The thymic medulla is composed of a dense network of distinct subsets of DCs and mTECs**. **(A)** Confocal micrograph of a mouse thymic section stained with antibodies against the DC-specific marker CD11c (blue) and the mTEC-specific marker K14 (red). **(B)** Three distinct subsets of DCs are located mainly in the medulla: resident cDCs (CD11c^hi^CD11b^−^ CD8α^hi^Sirpα^−^), migratory cDCs (CD11c^hi^CD11b^+^CD8α^lo^Sirpα^+^), and pDCs (CD11c^int^B220^+^PDCA-1^+^). **(C)** Schematic representation of mTEC differentiation. mTECs arise from a pool of self-renewing mTEC progenitors. Distinct stages of mTEC maturation can be identified based on the differential expression of MHCII, CD80, and Aire. The end stages of maturation can lead to the emergence of post-Aire mTECs, apoptosis, or to the development of Hassall’s corpuscle.

Healthy individuals mount effective T-cell immune responses directed against pathogens while avoiding autoimmune attacks directed toward self-antigens. The random generation of the T-cell receptor (TCR) repertoire results in the production of autoreactive TCRs, which necessitates their selection in the thymus (1). Anatomically, the thymus is compartmentalized into an outer region called the cortex and an inner region called the medulla. The cortex supports early stages of T-cell differentiation, including the positive selection of CD4^+^ and CD8^+^ thymocytes. Nonetheless, the cortex also supports a substantial loss of DP thymocytes that are specific for ubiquitous self-antigens (2, 3). The medulla sustains the induction of T-cell tolerance, which is established by two distinct main mechanisms: negative selection (also known as clonal deletion) of potentially hazardous autoreactive T cells, and the production of natural regulatory T (nTreg) cells. Negative selection consists of the deletion of immature T cells bearing TCRs, which are highly reactive against self-antigens (4, 5). Although this process is remarkably efficient, it cannot completely purge the TCR repertoire of self-reactive specificities and thus allows potentially hazardous T cells to reach the periphery. To control potential deleterious effects of autoreactive T cells that have escaped the negative selection process, the thymus produces a specific subset of T cells called nTregs. This cell type belongs mainly to the CD4^+^ T-cell lineage and specifically expresses the transcription factor forkhead box P3 (FOXP3), which is essential for their development and function (6). The induction of T-cell tolerance is established within the medullary microenvironment, which is composed of a dense 3D network of antigen-presenting cells (APCs), namely thymic dendritic cells (DCs) and medullary thymic epithelial cells (mTECs) (Figure 1A). In this review, we discuss our current knowledge regarding the phenotypic features of the different subsets of thymic DCs and mTECs as well as their relative contribution to the induction of T-cell tolerance. We also summarize recent progress in our understanding of the thymic crosstalk that sustains the composition and complex three-dimensional (3D) organization of the medulla.

## Thymic Medullary APCs Involved in T-Cell Tolerance Induction

### Features of thymic DCs

In the thymus, DCs represent only approximately 0.5% of the total thymic cells, which is less than that in other lymphoid organs. Although peripheral DCs have been long described as heterogeneous, only recently thymic DCs have also been shown to constitute a heterogeneous cell population. It is now accepted that thymic DCs comprise three distinct subsets: two conventional DC (cDC) subsets and plasmacytoid DCs (pDCs) (Figure 1B) (7). The two subsets of cDCs, which express high levels of CD11c, have different origins and can be distinguished based on specific cell surface markers. The CD11b^−^CD8α^hi^Sirpα^−^ (signal regulatory protein α) cDCs develop intrathymically and are commonly termed intrathymic or resident cDCs. In contrast, the CD11b^+^CD8α^lo^Sirpα^+^ cDCs have a myeloid origin and continuously migrate from the periphery via the blood circulation into the thymus (8). They are referred to as extrathymic or migratory cDCs. Under steady-state conditions, resident and migratory cDCs represent two-thirds and one-third of the thymic cDCs, respectively (7). Resident cDCs arise from a common T/DC precursor and reside exclusively in the thymus throughout their long life (7, 9, 10). They express CD8α mRNA and display CD8αα homodimers at their surface. In contrast, migratory cDCs do not synthesize CD8α mRNA, and the low expression level of CD8α observed at the surface of this cell type is a consequence of the uptake of cell surface CD8αβ heterodimers from thymocytes (11). Strikingly, following their migration in the thymus, migratory cDCs upregulate CD80 and CD86 costimulatory molecules as well as CD11c and MHCII molecules (8). In addition, in contrast to resident cDCs, migratory cDCs proliferate extensively and mature in interdigitating cDCs. Consequently, migratory cDCs overall exhibit a more activated phenotype compared with their resident counterparts (12).

The third subset of thymic DCs corresponds to pDCs, which continuously migrate to the thymus via the bloodstream. They are defined as CD11c^int^B220^+^PDCA-1^+^ and represent approximately 30% of the total thymic DCs (Figure 1B). Like their immature counterparts in the periphery, thymic pDCs present a plasmacytoid morphology rather than a dendritic morphology. Upon migration in the thymus, pDCs enlarge and adopt a semi-mature phenotype via the upregulation of CD11c and MHCII molecules (8). Moreover, they express high levels of Toll-like receptors (TLR) 7 and 9 and low levels of TLR 2, 3, and 4 (13). Overall, migratory DCs, i.e., pDCs and cDCs, represent 50% of the total thymic DCs. Parabiosis experiments have shown that migratory DCs are localized in the medulla and at the cortico-medullary junction (CMJ) (8). Antigen-loaded peripheral pDCs were found to be localized preferentially at the CMJ upon their migration in the thymus (14). Intriguingly, both pDCs and migratory cDCs change their phenotype shortly after entering the thymus, suggesting that the medullary microenvironment provides specific factors that contribute to the functional specification of these DC subsets. The identity of these factors that drive the maturation as well as the extensive proliferation of migratory DCs remains elusive.

### Features of mTECs

Similarly to DCs, mTECs also constitute a heterogeneous cell population that represent less than 1% of the total thymic cells (15). Histologically, mTECs are commonly identified by the expression of cytokeratin-5, 14, MTS10, and ERTR5 markers as well as by reactivity with the lectin Ulex Europaeus Agglutinin 1 (UEA-1) (16–19). However, it is not completely clear whether these markers stain the bulk of mTECs or whether they preferentially detect some specific subsets. However, the whole mTEC compartment can be identified by flow cytometry and is generally defined as CD45^−^EpCAM^+^ (epithelial cell adhesion molecule) Ly51^−/lo^. mTEC subsets can be further defined with respect to other markers, including the levels of cell surface MHCII and CD80 expression as well as of the transcription factor Aire (Figure 1C). Recent advances have established the relationship between these different cell subsets by demonstrating that mTEC differentiation proceeds along distinct maturational stages. RTOC experiments have shown that MHCII^−/lo^CD80^−/lo^Aire^−^immature mTECs give rise to MHCII^hi^CD80^hi^Aire^+^ mature mTECs (20–22). Consistently during embryogenesis, MHCII^−/lo^CD80^−/lo^Aire^−^immature mTECs appear prior to the emergence of MHCII^hi^CD80^hi^Aire^+^ mature mTECs (20, 22). Mature mTECs are thus believed to derive from immature mTECs via an intermediate stage that is Aire^−^ but has acquired high levels of MHCII and CD80 expression (Figure 1C). Aire^+^ mature mTECs were initially described to be post-mitotic and short-lived and were thus thought to represent the last stage of mTEC differentiation (20, 21). Apoptosis of this cell type has been proposed to be induced by Aire itself and to be favorable for the diffusion of self-antigens within the medullary microenvironment (21). Recent studies of cell fate mapping, allowing the permanent labeling of Aire-expressing cells even after the termination of transcription, have challenged this concept by demonstrating the existence of a post-Aire stage (23, 24). Approximately half of Aire^+^ mature mTECs seems to further progress to this post-Aire stage, which does not express Aire and expresses MHCII and CD80 molecules at reduced levels, thereby generating MHCII^lo^CD80^lo^Aire^−^mTECs (Figure 1C) (24, 25). This end-stage maturation of mTECs closely resembles that of keratinocytes (25). Finally, mTECs lose their nuclei to form Hassall’s corpuscles that can be detected by the expression of markers such as involucrin, cytokeratins 6/10, desmogleins 1/3, and lympho-epithelial kazal type related inhibitor (LEKTI) (25, 26).

Interestingly, all mTEC subsets are simultaneously present in the post-natal thymus (Figure 1C). In addition, the turnover period for mature mTECs is estimated to be between 2 and 3 weeks (20, 21). These observations suggest that the mature mTEC population is continuously replenished by differentiation from an mTEC progenitor. Consistent with this notion of perpetual renewal, recent studies have demonstrated the presence in adults of thymic epithelial progenitors and/or stem cells that are capable of generating both mature cortical and medullary lineages in a stepwise fashion (27, 28). Furthermore, a novel transitional progenitor stage characterized by the expression of cTEC markers such as CD205, β5t, and high levels of IL-7 has been identified in the embryonic thymus and shown to have the potential to generate mTECs (29–31). Moreover, an mTEC-specific stem cell capable of ensuring lifelong mTEC subsets was recently found within the claudin-3,4^hi^SSEA-1^+^ (stage-specific embryonic antigen 1) population (Figure 1C) (32). Of note, adult mTEC stem cells have a lower regenerative capacity than their embryonic counterparts. At the current stage of knowledge, the relationships among the common thymic epithelial stem cells (27, 28), the transitional progenitor that harbors cTEC-properties (29–31) and claudin-3,4^hi^ SSEA-1^+^ mTEC stem cells (32) remain unknown. Thus, further investigations are needed to clarify the relationship among these cells as well as their relative contributions to medulla formation and homeostasis within the embryonic and adult thymus. The identification of specific markers that allow distinct discrimination between these cell types would be helpful to evaluate their respective regenerative capacity. Such studies could aid in identifying clinical applications, notably for improving thymic function in the context of elderly or cytoablative treatments. Taken together, these findings have revealed that the medullary epithelium is not static but, in contrast, is much more dynamic than previously considered.

## Tight Collaboration between Medullary APCs for the Establishment of T-Cell Tolerance

Medullary thymic epithelial cells play a privileged role in the induction of central T-cell tolerance through their ability to express a broad range of tissue-restricted self-antigens (TRAs) (33). A recent study has shown, by using deep transcriptome sequencing, that mature mTECs express 19,293 genes, i.e., approximately 85% of the mouse genome (34). Thus, mTECs constitute the only cell type described that expresses such a large number of genes. The transcription factor Aire is the only regulator known to date that drives the expression of many TRAs (35). Aire alone regulates 3,980 genes (34). The importance of Aire in the induction of T-cell tolerance is illustrated by the fact that mutations in this gene are responsible for the development of the human autoimmune syndrome autoimmune polyendocrinopathy syndrome-1 (APS-1), which is also known as autoimmune polyendocrinopathy candidiasis ectodermal dystrophy (APECED) (36, 37). Similarly to humans, Aire-deficient mice show signs of autoimmunity characterized by inflammatory infiltrates and serum autoantibodies (38). The mechanisms by which Aire controls the transcription of TRAs have been extensively reviewed elsewhere (39–41). In contrast, although Aire-independent TRAs represent approximately 80% of the genes expressed in mTECs, the mechanisms that regulate them are largely unknown. The participation of other regulatory factors as well as epigenetic regulation thus remains to be identified.

### Cross-presentation of mTEC-derived TRAs by resident cDCs

**Figure 2:**
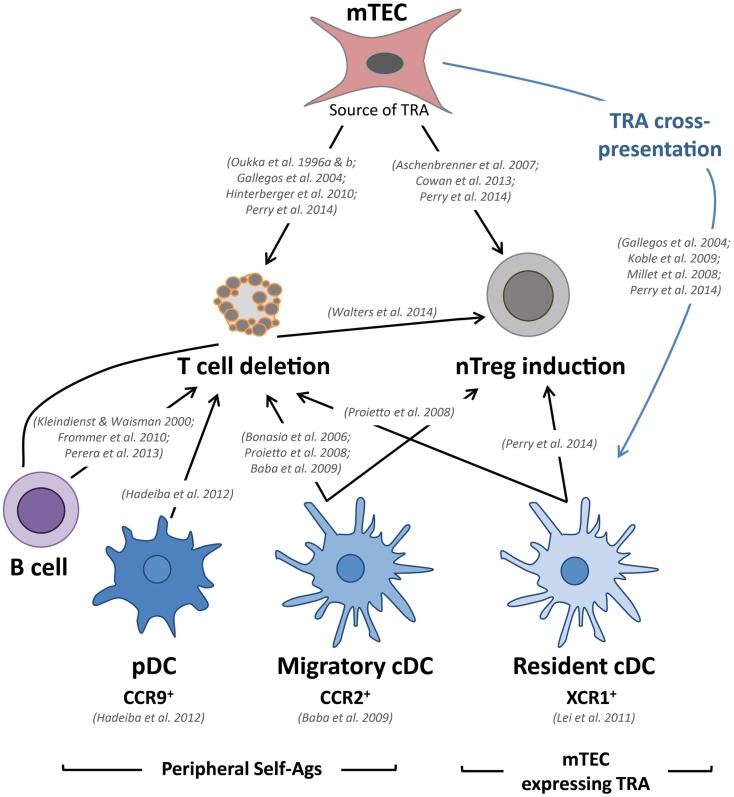
**mTECs and DCs tightly collaborate to delete autoreactive T cells and to induce the generation of nTreg cells**. Relevant *in vivo* studies are indicated in this figure. Tissue-restricted self-antigens (TRAs) expressed and presented by mTECs can lead to the deletion of autoreactive T cells and the induction of nTregs. These self-antigens can also be transferred to and presented by resident cDCs, resulting in T-cell deletion and the induction of nTregs. Furthermore, migratory cDCs and pDCs also reinforce the establishment of central T-cell tolerance via the presentation of antigens captured in the periphery. Migratory cDCs are also involved in T-cell deletion and the induction of nTregs, whereas pDCs only contribute to the deletion of autoreactive T cells in mice. Thymic B cells have also been shown to participate in the deletion of autoreactive T cells and the generation of nTregs.

Tissue-restricted self-antigens expressed by mTECs, independently of their subcellular origin, were described to be cross-presented by resident cDCs, which reside in close proximity to mTECs (42–45) (Figure 2). This unidirectional transfer of self-antigens is thought to be favored by a high mTEC turnover, which might allow the subsequent uptake of materials by cDCs. Although several potential mechanisms of intercellular material transfer have been proposed, such as the uptake of apoptotic bodies, gap junctions, exosome transfer, and membrane exchange (“nibbling”), experimental evidence is still lacking, and the precise underlying mechanisms remain unclear (46, 47). However, a recent study found that human TECs produce exosomes that carry antigen-presentation molecules and TRAs, suggesting that TEC-derived exosome could be involved in TRA cross-presentation (48). Given that a particular TRA is expressed only by a minor fraction of mTECs (1–3%), this phenomenon of intercellular antigen transfer likely ensures efficient scanning of TRAs by developing SP thymocytes (49). Furthermore, two-photon imaging experiments have shown that SP thymocytes are extremely mobile and make frequent and transient contacts with DCs, which might greatly contribute to the efficient selection of T cells during their 4- to 5-day residency in the medulla (50, 51). Proper localization of resident cDCs in the medulla is controlled by the expression of the chemokine receptor XCR1 and its Aire-dependent associated chemokine XCL1 (52). XCL1-deficient mice show fewer medullary DCs and defective generation of nTreg cells, suggesting that medullary cDCs contribute to nTreg cell development (Figure 2). Consistent with this observation, resident cDCs have been found to play an important role in the generation of nTregs via their ability to acquire and present Aire-dependent TRAs (53).

### mTECs act as *Bona Fide* APCs

Medullary thymic epithelial cells have thus been initially recognized to play a privileged role in T-cell tolerance because they constitute an “antigen reservoir” that mirrors the peripheral self (33). However, the use of transgenic mouse models that mimic TRA expression in the thymus have shown that mTECs can efficiently induce the clonal deletion of CD8^+^ T cells (42, 54). Recent studies have demonstrated that they also act as *bona fide* APCs to CD4^+^ T cells. mTECs have the ability to autonomously present endogenously expressed TRAs via MHCII molecules by using an unconventional endogenous pathway called macroautophagy, which allows the shuttling of cytoplasmic constituents into lysosomes (55, 56). Aire^+^ mTECs can induce both the negative selection of autoreactive T cells as well as the generation of nTreg cells (Figure 2) (53, 57–60). The induction of nTreg cells was found to be mTEC-dependent because mTECs have the ability to foster the development of Foxp3^−^CD25^+^ nTreg precursors (61). In accordance with these findings, mice showing an enhanced mTEC compartment display increased production of nTreg cells (62, 63). Conversely, mice showing a reduced mTEC compartment exhibit a reduction of nTreg cells (64, 65). Interestingly, a recent study has shown that a large proportion of thymic Tregs corresponds to peripheral recirculating Tregs (66). The participation of mTECs to this phenomenon of recirculation to the thymus remains to be examined. Interestingly, post-Aire mTECs were found to maintain intermediate TRA expression (24). Thus, it is plausible that this newly identified mTEC subset plays a role in the establishment of T-cell tolerance. Further studies, based for instance on cell-specific ablation, are needed to address this issue. Moreover, although MHCII^−/lo^CD80^−/lo^Aire^−^ and MHCII^hi^CD80^hi^Aire^−^ mTECs express fewer genes compared with Aire^+^ mTECs (34), only a few thousands genes are differentially expressed, which suggests that these immature subsets could have a non-redundant function in the induction of T-cell tolerance. In addition, these distinct mTEC subsets express different levels of MHCII and costimulatory molecules, which may significantly impact T-cell selection. Consistent with these observations, *in vivo* knock-down of MHCII molecules specifically in Aire^+^ mTECs leads to an increased proportion of CD4^+^ SP and an enhanced selection of nTregs (59). These findings suggest that there is an underlying division of labor within mTEC subsets, with immature mTECs likely providing more potent induction of nTregs and mature mTECs preferentially prone to negative selection. Of note, the *in vivo* dynamics of the interactions of CD8^+^ and CD4^+^ T cells with mTECs remain unknown to date. It would be very informative to compare the interactions of medullary CD8^+^ and CD4^+^ T cells with Aire^−^ and Aire^+^ mTECs to determine to what extent the frequency and duration of these interactions influence T-cell outcomes. Two-photon imaging experiments assessing fresh thymic slices are expected to achieve this goal in the near future and may reveal a complex choreography between SP thymocytes and mTECs.

### Migratory DCs reinforce the presentation of self-antigens

Although mTECs express a diverse repertoire of TRAs that largely contribute to the induction of T-cell tolerance, they cannot encompass the spectrum of all peripheral self-antigens. Migratory DCs have been shown to reinforce the deletion of autoreactive thymocytes by sampling peripheral self-antigens that would otherwise be undetectable to developing thymocytes. Studies based on Rag2^−/−^ OTII TCR-transgenic mice have shown that migratory cDCs induce the negative selection of autoreactive CD4^+^ thymocytes (12, 67). Interestingly, in co-culture assays, Sirpα^+^ cDCs efficiently convert CD4^+^CD25^−^ thymocytes into CD4^+^CD25^+^Foxp3^+^ nTregs (12, 68). Migratory cDCs were also found to efficiently induce nTreg cells *in vivo* (12). Thus, in the steady state, migratory cDCs have the ability to transport antigens captured in the periphery and contribute to the establishment of tolerance by deleting autoreactive CD4^+^ thymocytes and inducing nTreg cells (Figure 2). These studies have mainly focused on MHCII-restricted TCR-transgenic models, and consequently, the role of migratory cDCs in CD8^+^ T-cell deletion remains unclear. Migratory cDCs home to the thymus in a CCR2-dependent manner (69). CCR2-deficient mice display a decreased number of migratory cDCs in their thymus and exhibit defective negative selection against blood-borne antigens (69). However, the deficiency in CCR2 does not completely alter the migration of these cells, suggesting the potential involvement of other chemokine receptors. Of note, activated cDCs exhibit a reduced ability to home to the thymus, thus preventing the inappropriate deletion of cells capable of recognizing pathogen-derived antigens (67).

A third subset of DCs, namely pDCs, has recently been described to participate in the induction of T-cell tolerance. Until recently, the function of pDCs in the thymus has remained largely enigmatic, although it was suggested that they could protect the thymus against viral infections via their ability to produce type I interferon (7). In the periphery, in addition to secreting large amounts of type I interferon in response to viral infections, it became evident that pDCs can also function as *bona fide* APCs that are capable of modulating T-cell responses (70). Recent advances have demonstrated that pDCs possess tolerogenic properties in specific contexts, primarily through the induction or the proliferation of nTreg cells (71–74). Consistent with these tolerogenic functions observed in the periphery, pDCs were shown to colocalize with Foxp3^+^ Tregs and to promote the generation of nTreg cells from immature thymocytes via CD40–CD40L and interleukin-3 in the human thymus (75). Similarly, thymic stromal lymphopoietin (TSLP)-activated human pDCs induce the generation of nTregs (76). However, in mice, thymic pDCs do not efficiently induce the generation of nTregs from immature thymocytes *in vitro* (12, 68). *In vivo*, no role of thymic pDCs was observed in the conversion of thymocytes into the nTreg cell lineage (14). These studies suggest that in contrast to their human counterparts, murine thymic pDCs are intrinsically inefficient at inducing nTreg cells. Murine thymic pDCs, however, were shown to transport peripheral antigens to the thymus, inducing the deletion of autoreactive CD4^+^ thymocytes (14) (Figure 2). Their role in the deletion of CD8^+^ thymocytes remains unclear. The migration of pDCs in the thymus was found to be dependent on CCR9, a chemokine receptor that is also involved in T-cell progenitor homing (14, 77, 78). Importantly, pDCs that are activated by TLR ligands lose their ability to home to the thymus by down-regulating CCR9, thus preventing the unwanted induction of T-cell tolerance toward pathogens (14). Under normal conditions, CCR9 deficiency does not completely block the recruitment of pDCs in the thymus, suggesting that other chemokine receptors could be involved in this process. Interestingly, transgenic mice overexpressing CCL2 in the thymus under the myelin basic protein (MBP) promoter exhibit a massive thymic recruitment of pDCs, which express CCR2 (79, 80). The thymic migration of pDCs could be mediated via both CCR9 and CCR2. The generation of double knockout mice for CCR9 and CCR2 should reveal whether these two chemokine receptors are sufficient for directing the thymic recruitment of pDCs.

### A new player: Thymic B cells

In the medulla, in addition to mTECs and DCs, a third type of APC, namely the B cell, has also been implicated in the induction of T-cell tolerance (Figure 2). The vast majority of thymic B cells develop within the thymus from Rag-expressing progenitors, whereas recirculating B cells represent a minority (81). Thymic B cells display unique phenotypic hallmarks in comparison to peripheral B cells. They express high levels of MHCII and costimulatory molecules, supporting their robust antigen-presenting capacity (81). Of note, a recent report has shown that thymic B cells express Aire and display tolerogenic properties upon migration into the thymus (82). An original study using transgenic mice on an I-E-deficient background, in which B cells specifically express I-E MHCII molecules, established the capacity of thymic B cells to mediate the negative selection of CD4^+^ but not CD8^+^ T cells (83). Similarly, transgenic B cells, which exclusively present an antigen derived from the myelin oligodendrocyte glycoprotein (MOG), efficiently induce the deletion of MOG-specific CD4^+^ T cells (84). A recent study has also suggested that thymic B cells capture self-antigens through their B-cell receptors and delete autoreactive T cells by presenting peptides derived from these self-antigens (81). Furthermore, thymic B cells also contribute to the generation of nTreg cells (85).

Therefore, mTECs, DCs and B cells participate in the induction of T-cell tolerance through the negative selection of autoreactive T cells and the generation of nTreg cells (Figure 2). Interestingly, a recent study using deep sequencing in a fixed TCRβ chain model comparing different genetically modified mice has shown that bone marrow-derived APCs and mTECs play non-redundant roles in shaping the TCR repertoire (53). Roughly half of the Aire-dependent deletion or nTreg induction processes require antigen presentation by bone marrow cells (53). Moreover, the origin of the tissue antigens captured in the periphery and transported in the thymus by migratory cDCs and pDCs remains unclear. Additional studies are needed to determine the degree of the spectrum of overlap among antigens presented in the thymus by these two cell types. In addition to peripheral tissue antigens, although migratory DCs are suspected to participate in T-cell tolerance toward inoffensive foreign antigens derived from food or the commensal gut flora, experimental evidence is still lacking (86). Thus, it is possible that specific thymic DC subsets capture distinct sets of self-antigens and, consequently, could differentially impact the TCR repertoire. Additional studies performed at the polyclonal TCR level are required to elucidate this important issue.

## Involvement of Thymic Crosstalk in the Composition and Patterning of the Medulla

The thymic medulla plays a pivotal role in the selection of SP thymocytes. In turn, the expansion and organization of the medulla is governed by developing SP thymocytes. These reciprocal interactions between these two cell types is referred to as “thymic crosstalk” (87). Mice exhibiting a block in thymocyte development at the DP stage, such as TCRα^−/−^ and ZAP70^−/−^ mice, show prominent defects in medulla formation (88, 89). The transplantation of wild-type bone marrow cells in SCID mice lacking TCR-positive cells restores medulla formation and mTEC maturation, indicating that hematopoietic cells control the development of the medullary epithelium (90). Subsequent studies have established that TCR-bearing mature T cells control medulla formation (88, 91, 92). Thus, these pioneer studies indicated that SP thymocytes provide instructive signals that are critical for controlling the expansion and organization of the medulla. Recent advances have facilitated our understanding of the underlying molecular and cellular participants that are responsible for these crucial processes in the establishment of T-cell tolerance.

### AIRE^+^ mTEC differentiation in the embryonic thymus

**Figure 3:**
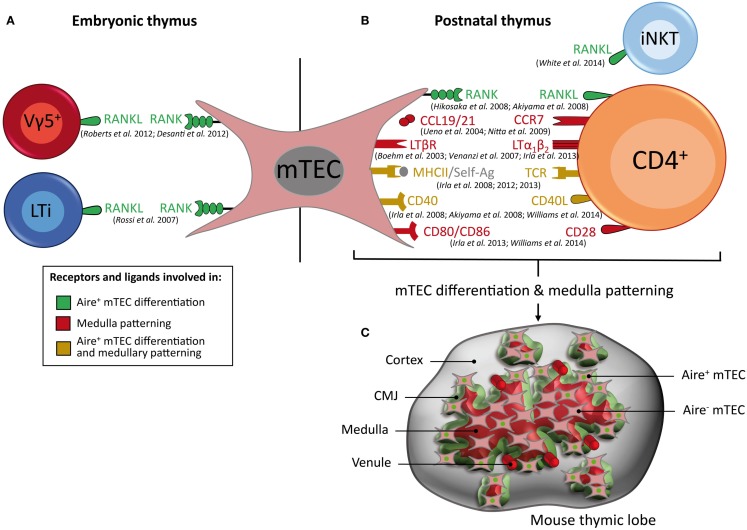
**Key cell types, receptors, and ligands that contribute to Aire^+^ mTEC differentiation and medulla patterning**. **(A)** RANKL, which is expressed by Vγ5^+^ T cells and LTi cells in the embryonic thymus and **(B)** by CD4^+^ thymocytes and iNKT cells in the post-natal thymus, induces Aire^+^ mTEC differentiation. In the post-natal thymus, crosstalk between mTECs and CD4^+^ thymocytes via CCL19/21–CCR7, LTβR/LTα1β2, and CD80/86–CD28 controls medulla patterning, whereas MHCII/self-antigen–TCR complexes and CD40–CD40L contribute to both the differentiation of Aire^+^ mTECs and patterning of the medulla. Receptors and ligands involved in Aire^+^ mTEC differentiation are represented in green, in medulla patterning in red, and in both processes in yellow. **(C)** Schematic representation of 3D medullary organization in the post-natal thymus. Aire^+^ mTECs (denoted by a green nucleus) and venules are preferentially localized at the cortico-medullary junction (CMJ).

In the embryonic thymus, lymphoid tissue inducer (LTi) cells identified as CD3^−^CD4^+^IL-7Rα^+^ were found to regulate the first cohort of Aire^+^ mTECs, which emerge around embryonic day 16 of gestation (Figure 3A) (20, 22, 93). LTi cells are present during embryogenesis at a time that correlates with the appearance of Aire^+^ mTECs, before the development of SP thymocytes (22). The emergence of Aire^+^ mTECs is controlled by a member of the tumor necrosis factor (TNF) superfamily: receptor activator of nuclear factor kappa-B (RANK), which is expressed by mTECs, and its corresponding ligand, RANKL (also known as TRANCE), which is expressed by LTi cells (Figure 3A). Strikingly, mice that are deficient for RANK or RANKL show an absence of Aire^+^ mTECs in the embryonic thymus, indicating that this TNF member regulates the emergence of Aire^+^ mTECs (22, 94). In accordance with these findings, the exposure of 2-deoxyguanosine-treated fetal thymus organ cultures (FTOCs) to recombinant RANKL or an agonistic antibody to RANK induces the appearance of mature mTECs (22, 95, 96). Conversely, the addition of osteoprotegerin, a soluble decoy receptor for RANKL, or the recombinant RANK-Fc protein, impairs Aire^+^ mTEC differentiation (94, 97). Importantly, LTi-deficient Rorc^−/−^ mice do not show a complete absence of Aire^+^ mTECs, suggesting that other embryonic cell types play a role in the development of the medullary epithelium (98). An additional cellular contributor, the invariant Vγ5^+^TCR^+^ dendritic epidermal T-cell progenitor, which also expresses RANKL, has likewise been recently implicated in the emergence of Aire^+^ mTECs in the embryonic thymus (Figure 3A) (97). The addition of purified Vγ5^+^ thymocytes or LTi cells in reaggregate thymus organ culture (RTOC) experiments induces similar proportions of Aire^+^ mTEC differentiation. Interestingly, Vγ5^+^ thymocytes and LTi cells are both present in individual Aire-expressing medullary environments, suggesting that they act collectively to influence mTEC maturation. Mice that are deficient in both LTi and γδ T cells (Rorc^−/−^ × Tcrd^−/−^mice) show a further decreased number of fetal Aire^+^ mTECs compared with mice that are deficient in either LTi or γδ T cells alone. However, Rorc^−/−^ × Tcrd^−/−^double-deficient mice do not show a complete absence of Aire^+^ mTECs, which suggests that other cell type(s) that remain(s) to be identified could also be involved in this differentiation process. Therefore, the two innate immune cells, Vγ5^+^ thymocytes and LTi cells, both of which express RANKL, drive the emergence of Aire^+^ mTECs in the embryonic thymus.

### AIRE^+^ mTEC differentiation in the post-natal thymus

In the post-natal thymus, the RANK–RANKL axis also plays a crucial role in the differentiation of mature mTECs (Figure 3B). The absence of RANK or RANKL expression leads to a drastic reduction of Aire^+^ mTECs and TRA expression (22, 94, 99). Conversely, mice that are deficient for osteoprotegerin, a soluble decoy receptor for RANKL, display a large medulla with many Aire-expressing mTECs (99). In contrast to the embryonic thymus, Aire^+^ mTECs are partially reduced in the post-natal thymus from RANK^−/−^ or RANKL^−/−^ mice, which suggests that after birth, additional signal(s) are involved in the differentiation and maintenance of mature mTECs. These observations led to the identification of a second member of the TNF superfamily, namely CD40, which is involved in this process (Figure 3B). CD40^−/−^ and CD40L^−/−^ mice show more subtle defects in mTEC subsets compared with those observed in RANK^−/−^ or RANKL^−/−^ mice (94, 100). However, these defects were markedly increased in RANK^−/−^ × CD40^−/−^double-deficient mice compared with RANK^−/−^mice, demonstrating that RANK and CD40 cooperate to promote mTEC differentiation in the post-natal thymus (94). Moreover, Aire and TRA expression are dramatically affected in these double-deficient mice, which consequently develop severe autoimmune manifestations. Taken together, these findings provide strong support for a model in which the emergence of Aire^+^ mTECs during embryogenesis involves RANK signaling, whereas the subsequent mTEC differentiation in the post-natal thymus involves cooperation between the RANK and CD40 signals (Figures 3A,B).

Several groups have investigated the cellular sources of RANKL and CD40L in the post-natal thymus. Although SP thymocytes were initially found to promote the organization and maturation of the medulla, it remained to be determined whether the instructive signals were provided in a different manner by CD4^+^ and/or CD8^+^ thymocytes. RANKL was found to be expressed by both CD4^+^ and CD8^+^ thymocytes, with a preferential expression by CD4^+^ thymocytes (101). In contrast, CD40L was found to be exclusively expressed by CD4^+^ thymocytes (99, 100). The simultaneous analysis of RANKL and CD40L proteins revealed a sequential acquisition of first RANKL on CD69^+^ semi-mature CD4^+^ thymocytes and then of CD40L on CD69^−^ mature CD4^+^ thymocytes, suggesting that RANKL and CD40L are delivered by distinct CD4^+^ subsets (101). The respective role of CD4^+^ and CD8^+^ thymocytes in mTEC differentiation was explicitly addressed through the use of knockout mice lacking either CD4^+^ or CD8^+^ thymocytes (100). The numbers of CD80^hi^Aire^+^ mature mTECs are essentially unaffected in mice lacking CD8^+^ thymocytes (β2m^−/−^ mice), which suggests that they are dispensable for this process. In contrast, the numbers of CD80^hi^Aire^+^ mature mTECs are strongly reduced in mice that lack the positive selection of CD4^+^ thymocytes, such as *H2-Aa*^−/−^ and *CIIta*^IV−/IV−^ mice (100). Thus, CD4^+^ thymocytes play a dominant role in promoting the development of the mature mTEC compartment (102). Nevertheless, in mice lacking CD4^+^ thymocytes, a minor population of CD80^hi^Aire^+^ mature mTECs is still detectable, suggesting that another cell type is also involved in the acquisition of a mature phenotype. Even if a rare number of LTi cells is present in the post-natal thymus, it is unlikely that these cells contribute significantly to mTEC differentiation after birth because Id2^−/−^ mice, which lack LTi cells, exhibit normal mature mTEC cellularity (99). Similarly, TCRγδ-deficient mice do not exhibit any obvious defect in mature mTECs (99). Thus, LTi and TCRγδ cells seem to be dispensable for mTEC differentiation during post-natal life. A recent study has suggested that invariant NKT cells that also express RANKL participate in Aire^+^ mTEC differentiation in adult mice (103). Thus, it is likely that CD4^+^ thymocytes and invariant NKT cells cooperate to drive mTEC differentiation (Figure 3B). Although CD4^+^ thymocytes play a dominant role in mTEC differentiation, it remained to be determined whether they influence this differentiation process via the release of soluble mediators or by directly engaging in physical interactions with mTECs. The generation of transgenic mice lacking MHCII molecules specifically in mTECs has shown that TCR–MHCII-mediated contacts between the two cell types are required for normal mature mTEC cellularity (100). Furthermore, mTEC differentiation occurs only when CD4^+^ thymocytes recognize their cognate antigen on mTECs (96, 100). Taken together, these findings revealed distinct molecular and cellular mechanisms that sustain the generation of mTECs that display a mature phenotype in the embryonic and post-natal thymus.

### Three-dimensional organization of the thymic medulla

The 3D reconstruction of wild-type thymic lobes has revealed that the medulla is highly complex, consisting of a major central compartment surrounded by ~100 islets (Figure 3C) (104–106). Interestingly, individual medullary islets initially derive from a single progenitor (107). Thus, during thymic development, some growing mTEC islets likely fuse together, leading to the emergence of larger islets and ultimately to a major central compartment. Additional studies of the 3D organization of the thymic lobes during thymus development from fetal to adult stage would be extremely informative to further understand the formation of the medullary architecture. Similar studies performed during aging should also reveal fundamental mechanisms of thymic involution. The recent development of multicolor fate mapping systems based on Cre-lox technology are expected to unravel the dynamics of the development and remodeling of the medulla during a lifetime (108). Importantly, such transgenic systems should aid in determining whether individual medullary islets are indeed derived from a single progenitor or, alternatively, whether they are derived from several clones. The discovery of the co-existence of a major medullary compartment and a hundred distinct smaller islets with a broad volume distribution raises questions regarding the functional relevance of individual islets compared to the central medulla. We estimate that individual islets may contain from only a few to as many as several thousand cells, with an average of a few tens or a few hundreds of cells (unpublished observations). Thus, for the smallest islets, it remains to be determined whether this low number of cells expresses a TRA array large enough to induce T-cell tolerance or, in contrast, whether these few cells do not express sufficient TRA and may permit the escape of potentially hazardous autoreactive T cells. It would be interesting to determine whether the large medulla and small individual islets display similar sets of TRAs. In addition, to be functional, small medullary islets must be vascularized, which remains to be further investigated using 3D reconstruction (106). Thus, further investigation is required to improve our understanding of the functional implications of the medullary topology. Of note, the medulla is not smooth at all, but on the contrary exhibits a highly folded/convoluted shape, with a complex contour at any scale, ranging from the total structure to the cellular level. Such multi-scale complexity is described best by fractal geometry, which affords a high area of interface for a given volume (105). Such characteristics are also typically found in the lungs or intestinal microvilli, which have a large surface area to maximize the exchange of oxygen or nutrients, respectively. In the case of the thymic medulla, this fractal shape ensures a large interface area between the cortex and the medulla, which is referred to as the CMJ. This fractal geometry also ensures that the average distance from any location within the cortex to the nearest medulla remains reasonably low (105). By comparison, the distances from any location within the cortex to the nearest medullary location are significantly reduced compared with the distances that would be obtained for the simplest shape, i.e., a spherical medulla. The CMJ likely plays a critical role in the function of the thymus because it constitutes the site where T cells go through at three critical steps of their journey through the thymus: (i) T-cell progenitors enter the thymus from venules preferentially located at the CMJ, travel outward in the cortex and subsequently migrate inward from the cortex to the medulla, undergoing positive selection; (ii) they cross the CMJ and migrate through the medulla, undergoing negative selection; (iii) they ultimately leave the thymus and enter the periphery, again via venules located at the CMJ (109–111). Indeed, the CMJ exhibits a high density of large venules, representing a privileged site for thymocyte homing/export, by extra/intravasation through venule walls, respectively (Figure 3C). Remarkably, the CMJ is also particularly dense in Aire^+^ mTECs, which is expected to favor the encounter with SP thymocytes that are migrating from the cortex to the medulla (93, 105). This distribution is strikingly pronounced in neonates compared to adults. This observation is consistent with the finding that Aire is important during the perinatal period to prevent the emergence of autoimmune disorders (112). Therefore, the CMJ represents not only a privileged site of T-cell progenitor homing and export of mature T cells but also a privileged region that favors the encounter of SP thymocytes with Aire^+^ mTECs. A first wave of negative selection is thus expected to occur in this region, which could play a more important role in the induction of T-cell tolerance than previously thought.

### Cellular and molecular crosstalk in medulla organization

Alterations in the cortico-medullary migration of SP thymocytes result in marked defects in the medullary organization. This is well illustrated in mice that lack CCR7 expressed by SP thymocytes or its two ligands CCL19 and CCL21 expressed by mTECs, which are responsible for the migration of SP thymocytes from the cortex to the medulla (113). CCR7- and CCR7 ligand-deficient mice show an arrest of thymocyte migration in the cortex and abnormal medulla formation characterized by small medullary regions that are sparsely distributed throughout their thymi (113, 114). The complex 3D organization of the medulla is preferentially controlled by positively selected CD4^+^ thymocytes (105, 115). H2-Aa^−/−^ mice lacking CD4^+^ thymocytes are devoid of any large medullary compartment, leading to a reduced medullary volume. In these mice, the numbers of mTECs are severely reduced, affecting CD80^hi^Aire^−^ and CD80^hi^Aire^+^ subsets (96). However, the formation of the medulla is less severely affected in mice lacking CD4^+^ thymocytes than in mice lacking SP thymocytes such as TCRα^−/−^ and ZAP70^−/−^ mice. These observations suggest that other cell type(s) participate in the expansion of the medulla. Although invariant NKT cells have been implicated in Aire^+^ mTEC differentiation, their role in the organization of the medulla remains to be defined. CD8^+^ thymocytes seem to play a minor role compared with CD4^+^ thymocytes because β2m^−/−^ mice, which lack CD8^+^ thymocytes, do not exhibit defects either in the 3D organization of the medulla or in the composition of the mTEC subset (96, 105). These observations suggest that CD4^+^ thymocytes are prominently required for the development and 3D organization of the medulla by controlling mTEC cellularity. Furthermore, the organization of the medulla is also dependent on antigen-specific TCR–MHCII-mediated interactions between autoreactive CD4^+^ thymocytes and mTECs displaying autoantigen–MHCII complexes (96). Several MHCII-restricted TCR-transgenic mice lacking expression of the cognate antigen, such as OTII-Rag2^−/−^, B3K508-Rag1^−/−^, and female Marilyn-Rag2^−/−^ mice, show severe impairment in medulla formation. In contrast, this defect is restored when the cognate antigen is expressed by mTECs, as for example in OTII-Rag2^−/−^ mice crossed with Rip-mOVA mice (in which the Rip-mOVA transgene drives the synthesis of membrane-bound OVA specifically in mTECs), or provided exogenously, as for example in OTII-Rag2^−/−^ mice injected with OVA_323–339_ peptide. Moreover, RTOC experiments in which OTII-Rag2^−/−^ thymocytes are reaggregated in the presence or absence of OVA_323–339_ peptide have demonstrated that the addition of the cognate antigen restores the numbers of mTECs similarly to those induced by WT thymocytes (96). These antigen-specific interactions between mTECs and CD4^+^ thymocytes also require the engagement of the CD28–CD80/86 and CD40–CD40L costimulatory axes (Figure 3B). Defects in the CD28–CD80/86 or CD40–CD40L costimulatory pathway alone have a slight effect on the architecture of the medulla (105, 116). In contrast, the combined absence of CD28–CD80/86 and CD40–CD40L results in a drastic impairment in medulla formation (116). These different experimental results thus favor a model in which autoreactive CD4^+^ thymocytes control the formation and organization of the medulla in an antigen-dependent manner that involves the CD28–CD80/86 and CD40–CD40L costimulatory pathways. Interestingly, two-photon microscopy experiments have revealed that autoreactive thymocytes do not directly undergo cell death after encountering a negative selecting ligand but instead remain viable and motile for some time in the medullary microenvironment (51). They adopt a confined migration pattern during which they likely provide to mTECs instructive signals that would be necessary for both mTEC differentiation and organization.

A third member of the TNF superfamily, namely the lymphotoxin β receptor (LTβR) expressed by mTECs, and its ligand, the heterotrimer LTα1β2 expressed by SP thymocytes, was found to orchestrate the organization of the medulla (105, 117, 118). A deficiency in LTβR leads to a disorganized medullary architecture and alterations in mTEC subsets, notably in UEA-1^+^ mTECs and terminally differentiated involucrin^+^ mTECs (117–119). LTβR signaling also regulates the expression of Aire-independent TRAs and CCL19 in mTECs (120, 121). These defects are associated with the appearance of signs of autoimmunity, which suggests that LTβR signaling is required for the establishment of central tolerance (117, 120). Of note, mice that are deficient for LTβR ligands, such as LTα^−/−^, LTβ^−/−^, or LIGHT^−/−^ mice, exhibit an intermediate phenotype compared with that observed in LTβR^−/−^ mice. Consequently, the contribution of lymphotoxin signaling to mTEC development is only partially understood (117, 118). The 3D reconstruction of LTα^−/−^ thymic lobes has revealed that LTα^−/−^ mice are devoid of any large medullary compartment, leading to a substantial reduction of the medulla volume. Of note, the absence of LTα results in a less drastic phenotype compared with that observed in mice lacking CD4^+^ thymocytes, which suggests that other(s) mediator(s) contribute to the effect mediated by CD4^+^ T cells (105). Interestingly, the absence of Aire results in morphological changes in mTECs (26). However, it remains unclear whether Aire affects the 3D organization of the medulla in terms of the numbers and volumes of the medullary islets. Further investigations including the identification of other molecular participants in the topology of the medulla as well as the determination of the 3D distribution of specific mTEC subsets are required. Indeed, recent findings have revealed a differential distribution of mTEC subsets throughout the medulla. Aire^+^ mTECs were found to be preferentially positioned at the CMJ, whereas post-Aire mTECs were described to be localized toward the center of the medulla (24, 93, 105). A 3D map of distinct mTEC subsets, including mTEC stem cells, may thus reveal a subtle compartmentalization of these specific cell types within the thymic medulla.

Importantly, this cellular crosstalk between mTECs and autoreactive CD4^+^ thymocytes regulates a cascade of events that control the expression of TNF superfamily members that are essential for both the differentiation and organization of mTECs. In this context, autoantigen-specific interactions between mTECs and CD4^+^ thymocytes, involving the CD40–CD40L and CD28–CD80/86 axes, lead to the upregulation of lymphotoxin ligands in autoreactive CD4^+^ thymocytes (96, 116). Then, LTβR signaling induces RANK expression in mTECs (95, 96), and subsequently, RANK signaling induces the upregulation of CD40 in mTECs (101). This cellular crosstalk with autoreactive CD4^+^ thymocytes is likely to fine-tune the homeostasis of the medulla, allowing the thymus to adapt optimally for the establishment of T-cell tolerance.

## Concluding Remarks

Thymic crosstalk is the indispensable interplay between medullary APCs and developing T cells that coordinates the induction of T-cell tolerance. DCs, B cells, and mTECs have all been shown to control the selection of SP thymocytes. DCs reinforce the induction of T-cell tolerance by cross-presenting mTEC-derived TRAs and by displaying peripheral self-antigens captured in the periphery. Furthermore, thymic B cells can also express Aire and act as APCs. Nevertheless, mTECs are the lead player in T-cell tolerance induction due to their constitutive expression of TRAs. At the molecular and cellular levels, studies conducted over the last decade have furthered our understanding of the thymic crosstalk that sustains mTEC differentiation as well as the organization of the medulla. However, the precise consequences of thymic crosstalk on mTEC differentiation, proliferation, and survival remain to be defined. Additional studies are needed to identify the downstream target genes induced in mTECs by crosstalk signals in both the embryonic and the post-natal thymus. Future work can be expected to elucidate how thymic crosstalk shapes the T cell repertoire. Such studies would be extremely informative for further delineating the mechanisms that govern the establishment of T-cell tolerance. This knowledge is expected to pave the way toward novel therapeutic strategies aimed at preventing the development of autoimmunity and controlling age-associated thymic involution.

## Conflict of Interest Statement

The authors declare that the research was conducted in the absence of any commercial or financial relationships that could be construed as a potential conflict of interest.

## References

[1] Palmer E. Negative selection – clearing out the bad apples from the T-cell repertoire. Nat Rev Immunol (2003) 3:383–91. 10.1038/nri1085 12766760

[2] McCaughtry TM Baldwin TA Wilken MS Hogquist KA. Clonal deletion of thymocytes can occur in the cortex with no involvement of the medulla. J Exp Med (2008) 205:2575–84. 10.1084/jem.20080866 18936237 PMC2571932

[3] Stritesky GL Xing Y Erickson JR Kalekar LA Wang X Mueller DL Murine thymic selection quantified using a unique method to capture deleted T cells. Proc Natl Acad Sci U S A (2013) 110:4679–84. 10.1073/pnas.1217532110 23487759 PMC3606987

[4] Kappler JW Roehm N Marrack P. T cell tolerance by clonal elimination in the thymus. Cell (1987) 49:273–80. 10.1016/0092-8674(87)90568-X 3494522

[5] Pircher H Burki K Lang R Hengartner H Zinkernagel RM. Tolerance induction in double specific T-cell receptor transgenic mice varies with antigen. Nature (1989) 342:559–61. 10.1038/342559a0 2573841

[6] Josefowicz SZ Lu LF Rudensky AY. Regulatory T cells: mechanisms of differentiation and function. Annu Rev Immunol (2012) 30:531–64. 10.1146/annurev.immunol.25.022106.141623 22224781 PMC6066374

[7] Wu L Shortman K Heterogeneity of thymic dendritic cells. Semin Immunol (2005) 17:304–12. 10.1016/j.smim.2005.05.001 15946853

[8] Li J Park J Foss D Goldschneider I. Thymus-homing peripheral dendritic cells constitute two of the three major subsets of dendritic cells in the steady-state thymus. J Exp Med (2009) 206:607–22. 10.1084/jem.20082232 19273629 PMC2699131

[9] Ardavin C Wu L Li CL Shortman K. Thymic dendritic cells and T cells develop simultaneously in the thymus from a common precursor population. Nature (1993) 362:761–3. 10.1038/362761a0 8469288

[10] Porritt HE Gordon K Petrie HT. Kinetics of steady-state differentiation and mapping of intrathymic-signaling environments by stem cell transplantation in nonirradiated mice. J Exp Med (2003) 198:957–62. 10.1084/jem.20030837 12975459 PMC2194209

[11] Vremec D Pooley J Hochrein H Wu L Shortman K. CD4 and CD8 expression by dendritic cell subtypes in mouse thymus and spleen. J Immunol (2000) 164:2978–86. 10.4049/jimmunol.164.6.2978 10706685

[12] Proietto AI Van Dommelen S Zhou P Rizzitelli A D’amico A Steptoe RJ Dendritic cells in the thymus contribute to T-regulatory cell induction. Proc Natl Acad Sci U S A (2008) 105:19869–74. 10.1073/pnas.0810268105 19073916 PMC2604962

[13] Okada T Lian ZX Naiki M Ansari AA Ikehara S Gershwin ME. Murine thymic plasmacytoid dendritic cells. Eur J Immunol (2003) 33:1012–9. 10.1002/eji.200323616 12672067

[14] Hadeiba H Lahl K Edalati A Oderup C Habtezion A Pachynski R Plasmacytoid dendritic cells transport peripheral antigens to the thymus to promote central tolerance. Immunity (2012) 36:438–50. 10.1016/j.immuni.2012.01.017 22444632 PMC3315699

[15] Gray DH Fletcher AL Hammett M Seach N Ueno T Young LF Unbiased analysis, enrichment and purification of thymic stromal cells. J Immunol Methods (2008) 329:56–66. 10.1016/j.jim.2007.09.010 17988680

[16] Van Vliet E Melis M Van Ewijk W. Monoclonal antibodies to stromal cell types of the mouse thymus. Eur J Immunol (1984) 14:524–9. 10.1002/eji.1830140608 6734714

[17] Farr AG Anderson SK. Epithelial heterogeneity in the murine thymus: fucose-specific lectins bind medullary epithelial cells. J Immunol (1985) 134:2971–7. 3856612

[18] Godfrey DI Izon DJ Tucek CL Wilson TJ Boyd RL The phenotypic heterogeneity of mouse thymic stromal cells. Immunology (1990) 70:66–74. 2191917 PMC1384083

[19] Klug DB Carter C Crouch E Roop D Conti CJ Richie ER. Interdependence of cortical thymic epithelial cell differentiation and T-lineage commitment. Proc Natl Acad Sci U S A (1998) 95:11822–7. 10.1073/pnas.95.20.11822 9751749 PMC21724

[20] Gabler J Arnold J Kyewski B. Promiscuous gene expression and the developmental dynamics of medullary thymic epithelial cells. Eur J Immunol (2007) 37:3363–72. 10.1002/eji.200737131 18000951

[21] Gray D Abramson J Benoist C Mathis D. Proliferative arrest and rapid turnover of thymic epithelial cells expressing Aire. J Exp Med (2007) 204:2521–8. 10.1084/jem.20070795 17908938 PMC2118482

[22] Rossi SW Kim MY Leibbrandt A Parnell SM Jenkinson WE Glanville SH RANK signals from CD4(+)3(-) inducer cells regulate development of Aire-expressing epithelial cells in the thymic medulla. J Exp Med (2007) 204:1267–72. 10.1084/jem.20062497 17502664 PMC2118623

[23] Nishikawa Y Hirota F Yano M Kitajima H Miyazaki J Kawamoto H Biphasic Aire expression in early embryos and in medullary thymic epithelial cells before end-stage terminal differentiation. J Exp Med (2010) 207:963–71. 10.1084/jem.20092144 20404099 PMC2867289

[24] Metzger TC Khan IS Gardner JM Mouchess ML Johannes KP Krawisz AK Lineage tracing and cell ablation identify a post-Aire-expressing thymic epithelial cell population. Cell Rep (2013) 5:166–79. 10.1016/j.celrep.2013.08.038 24095736 PMC3820422

[25] Wang X Laan M Bichele R Kisand K Scott HS Peterson P. Post-Aire maturation of thymic medullary epithelial cells involves selective expression of keratinocyte-specific autoantigens. Front Immunol (2012) 3:19. 10.3389/fimmu.2012.00019 22448160 PMC3310317

[26] Yano M Kuroda N Han H Meguro-Horike M Nishikawa Y Kiyonari H Aire controls the differentiation program of thymic epithelial cells in the medulla for the establishment of self-tolerance. J Exp Med (2008) 205:2827–38. 10.1084/jem.20080046 19015306 PMC2585853

[27] Ucar A Ucar O Klug P Matt S Brunk F Hofmann TG Adult thymus contains FoxN1(-) epithelial stem cells that are bipotent for medullary and cortical thymic epithelial lineages. Immunity (2014) 41:257–69. 10.1016/j.immuni.2014.07.005 25148026 PMC4148705

[28] Wong K Lister NL Barsanti M Lim JM Hammett MV Khong DM Multilineage potential and self-renewal define an epithelial progenitor cell population in the adult thymus. Cell Rep (2014) 8:1198–209. 10.1016/j.celrep.2014.07.029 25131206

[29] Baik S Jenkinson EJ Lane PJ Anderson G Jenkinson WE. Generation of both cortical and Aire(+) medullary thymic epithelial compartments from CD205(+) progenitors. Eur J Immunol y (2013) 43:589–94. 10.1002/eji.201243209 23299414 PMC3960635

[30] Ohigashi I Zuklys S Sakata M Mayer CE Zhanybekova S Murata S Aire-expressing thymic medullary epithelial cells originate from beta5t-expressing progenitor cells. Proc Natl Acad Sci U S A (2013) 110:9885–90. 10.1073/pnas.1301799110 23720310 PMC3683726

[31] Ribeiro AR Rodrigues PM Meireles C Di Santo JP Alves NL. Thymocyte selection regulates the homeostasis of IL-7-expressing thymic cortical epithelial cells in vivo. J Immunol (2013) 191:1200–9. 10.4049/jimmunol.1203042 23794633

[32] Sekai M Hamazaki Y Minato N. Medullary thymic epithelial stem cells maintain a functional thymus to ensure lifelong central T Cell tolerance. Immunity (2014) 41:753–61. 10.1016/j.immuni.2014.10.011 25464854

[33] Derbinski J Schulte A Kyewski B Klein L. Promiscuous gene expression in medullary thymic epithelial cells mirrors the peripheral self. Nat Immunol (2001) 2:1032–9. 10.1038/ni723 11600886

[34] Sansom SN Shikama-Dorn N Zhanybekova S Nusspaumer G Macaulay IC Deadman ME Population and single-cell genomics reveal the Aire dependency, relief from polycomb silencing, and distribution of self-antigen expression in thymic epithelia. Genome Res (2014) 24(12):1918–31. 10.1101/gr.171645.113 25224068 PMC4248310

[35] Anderson MS Venanzi ES Klein L Chen Z Berzins SP Turley SJ Projection of an immunological self shadow within the thymus by the Aire protein. Science (2002) 298:1395–401. 10.1126/science.1075958 12376594

[36] Consortium F-GA. An autoimmune disease, APECED, caused by mutations in a novel gene featuring two PHD-type zinc-finger domains. Nat Genet (1997) 17:399–403. 10.1038/ng1297-399 9398840

[37] Nagamine K Peterson P Scott HS Kudoh J Minoshima S Heino M Positional cloning of the APECED gene. Nat Genet (1997) 17:393–8. 10.1038/ng1297-393 9398839

[38] Jiang W Anderson MS Bronson R Mathis D Benoist C. Modifier loci condition autoimmunity provoked by Aire deficiency. J Exp Med (2005) 202:805–15. 10.1084/jem.20050693 16172259 PMC2212943

[39] Peterson P Org T Rebane A. Transcriptional regulation by AIRE: molecular mechanisms of central tolerance. Nat Rev Immunol (2008) 8:948–57. 10.1038/nri2450 19008896 PMC2785478

[40] Akiyama T Shinzawa M Qin J Akiyama N. Regulations of gene expression in medullary thymic epithelial cells required for preventing the onset of autoimmune diseases. Front Immunol (2013) 4:249. 10.3389/fimmu.2013.00249 23986760 PMC3752772

[41] Zumer K Saksela K Peterlin BM. The mechanism of tissue-restricted antigen gene expression by AIRE. J Immunol (2013) 190:2479–82. 10.4049/jimmunol.1203210 23456700

[42] Gallegos AM Bevan MJ. Central tolerance to tissue-specific antigens mediated by direct and indirect antigen presentation. J Exp Med (2004) 200:1039–49. 10.1084/jem.20041457 15492126 PMC2211843

[43] Millet V Naquet P Guinamard RR. Intercellular MHC transfer between thymic epithelial and dendritic cells. Eur J Immunol (2008) 38:1257–63. 10.1002/eji.200737982 18412162

[44] Koble C Kyewski B. The thymic medulla: a unique microenvironment for intercellular self-antigen transfer. J Exp Med (2009) 206:1505–13. 10.1084/jem.20082449 19564355 PMC2715082

[45] Hubert FX Kinkel SA Davey GM Phipson B Mueller SN Liston A Aire regulates the transfer of antigen from mTECs to dendritic cells for induction of thymic tolerance. Blood (2011) 118:2462–72. 10.1182/blood-2010-06-286393 21505196

[46] Klein L Hinterberger M Wirnsberger G Kyewski B. Antigen presentation in the thymus for positive selection and central tolerance induction. Nat Rev Immunol (2009) 9:833–44. 10.1038/nri2669 19935803

[47] Klein L Hinterberger M Von Rohrscheidt J Aichinger M. Autonomous versus dendritic cell-dependent contributions of medullary thymic epithelial cells to central tolerance. Trends Immunol (2011) 32:188–93. 10.1016/j.it.2011.03.002 21493141

[48] Skogberg G Lundberg V Berglund M Gudmundsdottir J Telemo E Lindgren S Human thymic epithelial primary cells produce exosomes carrying tissue-restricted antigens. Immunol Cell Biol (2015). 10.1038/icb.2015.33 25776846 PMC4575951

[49] Klein L Kyewski B Allen PM Hogquist KA. Positive and negative selection of the T cell repertoire: what thymocytes see (and don’t see). Nat Rev Immunol (2014) 14:377–91. 10.1038/nri3667 24830344 PMC4757912

[50] McCaughtry TM Wilken MS Hogquist KA. Thymic emigration revisited. J Exp Med (2007) 204:2513–20. 10.1084/jem.20070601 17908937 PMC2118501

[51] Le Borgne M Ladi E Dzhagalov I Herzmark P Liao YF Chakraborty AK The impact of negative selection on thymocyte migration in the medulla. Nat Immunol (2009) 10:823–30. 10.1038/ni.1761 19543275 PMC2793676

[52] Lei Y Ripen AM Ishimaru N Ohigashi I Nagasawa T Jeker LT Aire-dependent production of XCL1 mediates medullary accumulation of thymic dendritic cells and contributes to regulatory T cell development. J Exp Med (2011) 208:383–94. 10.1084/jem.20102327 21300913 PMC3039864

[53] Perry JS Lio CW Kau AL Nutsch K Yang Z Gordon JI Distinct contributions of Aire and antigen-presenting-cell subsets to the generation of self-tolerance in the thymus. Immunity (2014) 41:414–26. 10.1016/j.immuni.2014.08.007 25220213 PMC4175925

[54] Oukka M Cohen-Tannoudji M Tanaka Y Babinet C Kosmatopoulos K. Medullary thymic epithelial cells induce tolerance to intracellular proteins. J Immunol (1996) 156:968–75. 8558024

[55] Nedjic J Aichinger M Emmerich J Mizushima N Klein L. Autophagy in thymic epithelium shapes the T-cell repertoire and is essential for tolerance. Nature (2008) 455:396–400. 10.1038/nature07208 18701890

[56] Aichinger M Wu C Nedjic J Klein L. Macroautophagy substrates are loaded onto MHC class II of medullary thymic epithelial cells for central tolerance. J Exp Med (2013) 210:287–300. 10.1084/jem.20122149 23382543 PMC3570095

[57] Oukka M Colucci-Guyon E Tran PL Cohen-Tannoudji M Babinet C Lotteau V CD4 T cell tolerance to nuclear proteins induced by medullary thymic epithelium. Immunity (1996) 4:545–53. 10.1016/S1074-7613(00)80481-1 8673701

[58] Aschenbrenner K D’cruz LM Vollmann EH Hinterberger M Emmerich J Swee LK Selection of Foxp3+ regulatory T cells specific for self antigen expressed and presented by Aire+ medullary thymic epithelial cells. Nat Immunol (2007) 8:351–8. 10.1038/ni1444 17322887

[59] Hinterberger M Aichinger M Da Costa OP Voehringer D Hoffmann R Klein L. Autonomous role of medullary thymic epithelial cells in central CD4(+) T cell tolerance. Nat Immunol (2010) 11:512–9. 10.1038/ni.1874 20431619

[60] Malchow S Leventhal DS Nishi S Fischer BI Shen L Paner GP Aire-dependent thymic development of tumor-associated regulatory T cells. Science (2013) 339:1219–24. 10.1126/science.1233913 23471412 PMC3622085

[61] Cowan JE Parnell SM Nakamura K Caamano JH Lane PJ Jenkinson EJ The thymic medulla is required for Foxp3+ regulatory but not conventional CD4+ thymocyte development. J Exp Med (2013) 210:675–81. 10.1084/jem.20122070 23530124 PMC3620359

[62] Akiyama N Shinzawa M Miyauchi M Yanai H Tateishi R Shimo Y Limitation of immune tolerance-inducing thymic epithelial cell development by Spi-B-mediated negative feedback regulation. J Exp Med (2014) 211:2425–38. 10.1084/jem.20141207 25385757 PMC4235644

[63] Hauri-Hohl M Zuklys S Hollander GA Ziegler SF. A regulatory role for TGF-beta signaling in the establishment and function of the thymic medulla. Nat Immunol (2014) 15:554–61. 10.1038/ni.2869 24728352

[64] Kajiura F Sun S Nomura T Izumi K Ueno T Bando Y NF-kappa B-inducing kinase establishes self-tolerance in a thymic stroma-dependent manner. J Immunol (2004) 172:2067–75. 10.4049/jimmunol.172.4.2067 14764671

[65] Mouri Y Nishijima H Kawano H Hirota F Sakaguchi N Morimoto J NF-kappaB-inducing kinase in thymic stroma establishes central tolerance by orchestrating cross-talk with not only thymocytes but also dendritic cells. J Immunol (2014) 193:4356–67. 10.4049/jimmunol.1400389 25261487

[66] Thiault N Darrigues J Adoue V Gros M Binet B Perals C Peripheral regulatory T lymphocytes recirculating to the thymus suppress the development of their precursors. Nat Immunol (2015) 16(6):628–34. 10.1038/ni.3150 25939024

[67] Bonasio R Scimone ML Schaerli P Grabie N Lichtman AH Von Andrian UH. Clonal deletion of thymocytes by circulating dendritic cells homing to the thymus. Nat Immunol (2006) 7:1092–100. 10.1038/ni1106-1234b 16951687

[68] Guerri L Peguillet I Geraldo Y Nabti S Premel V Lantz O. Analysis of APC types involved in CD4 tolerance and regulatory T cell generation using reaggregated thymic organ cultures. J Immunol (2013) 190:2102–10. 10.4049/jimmunol.1202883 23365074

[69] Baba T Nakamoto Y Mukaida N. Crucial contribution of thymic Sirp alpha+ conventional dendritic cells to central tolerance against blood-borne antigens in a CCR2-dependent manner. J Immunol (2009) 183:3053–63. 10.4049/jimmunol.0900438 19675159

[70] Villadangos JA Young L. Antigen-presentation properties of plasmacytoid dendritic cells. Immunity (2008) 29:352–61. 10.1016/j.immuni.2008.09.002 18799143

[71] Ito T Yang M Wang YH Lande R Gregorio J Perng OA Plasmacytoid dendritic cells prime IL-10-producing T regulatory cells by inducible costimulator ligand. J Exp Med (2007) 204:105–15. 10.1084/jem.20061660 17200410 PMC2118437

[72] Sharma MD Baban B Chandler P Hou DY Singh N Yagita H Plasmacytoid dendritic cells from mouse tumor-draining lymph nodes directly activate mature Tregs via indoleamine 2,3-dioxygenase. J Clin Invest (2007) 117:2570–82. 10.1172/JCI31911 17710230 PMC1940240

[73] Hadeiba H Sato T Habtezion A Oderup C Pan J Butcher EC. CCR9 expression defines tolerogenic plasmacytoid dendritic cells able to suppress acute graft-versus-host disease. Nat Immunol (2008) 9:1253–60. 10.1038/ni.1658 18836452 PMC2901237

[74] Irla M Kupfer N Suter T Lissilaa R Benkhoucha M Skupsky J MHC class II-restricted antigen presentation by plasmacytoid dendritic cells inhibits T cell-mediated autoimmunity. J Exp Med (2010) 207:1891–905. 10.1084/jem.20092627 20696698 PMC2931160

[75] Martin-Gayo E Sierra-Filardi E Corbi AL Toribio ML. Plasmacytoid dendritic cells resident in human thymus drive natural Treg cell development. Blood (2010) 115:5366–75. 10.1182/blood-2009-10-248260 20357241

[76] Hanabuchi S Ito T Park WR Watanabe N Shaw JL Roman E Thymic stromal lymphopoietin-activated plasmacytoid dendritic cells induce the generation of FOXP3+ regulatory T cells in human thymus. J Immunol (2010) 184:2999–3007. 10.4049/jimmunol.0804106 20173030 PMC3325785

[77] Krueger A Willenzon S Lyszkiewicz M Kremmer E Forster R. CC chemokine receptor 7 and 9 double-deficient hematopoietic progenitors are severely impaired in seeding the adult thymus. Blood (2010) 115:1906–12. 10.1182/blood-2009-07-235721 20040757

[78] Zlotoff DA Sambandam A Logan TD Bell JJ Schwarz BA Bhandoola A. CCR7 and CCR9 together recruit hematopoietic progenitors to the adult thymus. Blood (2010) 115:1897–905. 10.1182/blood-2009-08-237784 19965655 PMC2837318

[79] Proietto AI Van Dommelen S Wu L. The impact of circulating dendritic cells on the development and differentiation of thymocytes. Immunol Cell Biol (2009) 87:39–45. 10.1038/icb.2008.86 19048018

[80] Cedile O Lobner M Toft-Hansen H Frank I Wlodarczyk A Irla M Thymic CCL2 influences induction of T-cell tolerance. J Autoimmun (2014) 55:73–85. 10.1016/j.jaut.2014.07.004 25129504

[81] Perera J Meng L Meng F Huang H. Autoreactive thymic B cells are efficient antigen-presenting cells of cognate self-antigens for T cell negative selection. Proc Natl Acad Sci U S A (2013) 110:17011–6. 10.1073/pnas.1313001110 24082098 PMC3801014

[82] Yamano T Nedjic J Hinterberger M Steinert M Koser S Pinto S Thymic B cells are licensed to present self antigens for central T cell tolerance induction. Immunity (2015) 42(6):1048–61. 10.1016/j.immuni.2015.05.013 26070482

[83] Kleindienst P Chretien I Winkler T Brocker T. Functional comparison of thymic B cells and dendritic cells in vivo. Blood (2000) 95:2610–6. 10753841

[84] Frommer F Waisman A. B cells participate in thymic negative selection of murine auto-reactive CD4+ T cells. PLoS One (2010) 5:e15372. 10.1371/journal.pone.0015372 20976010 PMC2958132

[85] Walters SN Webster KE Daley S Grey ST. A role for intrathymic B cells in the generation of natural regulatory T cells. J Immunol (2014) 193:170–6. 10.4049/jimmunol.1302519 24872190

[86] Derbinski J Kyewski B. How thymic antigen presenting cells sample the body’s self-antigens. Curr Opin Immunol (2010) 22:592–600. 10.1016/j.coi.2010.08.003 20832274

[87] van Ewijk W Shores EW Singer A Crosstalk in the mouse thymus. Immunol Today (1994) 15:214–7. 10.1016/0167-5699(94)90246-1 8024681

[88] Palmer DB Viney JL Ritter MA Hayday AC Owen MJ. Expression of the alpha beta T-cell receptor is necessary for the generation of the thymic medulla. Dev Immunol (1993) 3:175–9. 10.1155/1993/56290 8281032 PMC2275932

[89] Negishi I Motoyama N Nakayama K Nakayama K Senju S Hatakeyama S Essential role for ZAP-70 in both positive and negative selection of thymocytes. Nature (1995) 376:435–8. 10.1038/376435a0 7630421

[90] Shores EW Van Ewijk W Singer A. Disorganization and restoration of thymic medullary epithelial cells in T cell receptor-negative scid mice: evidence that receptor-bearing lymphocytes influence maturation of the thymic microenvironment. Eur J Immunol (1991) 21:1657–61. 10.1002/eji.1830210711 2060577

[91] Surh CD Ernst B Sprent J. Growth of epithelial cells in the thymic medulla is under the control of mature T cells. J Exp Med (1992) 176:611–6. 10.1084/jem.176.2.611 1500862 PMC2119324

[92] Shores EW Van Ewijk W Singer A. Maturation of medullary thymic epithelium requires thymocytes expressing fully assembled CD3-TCR complexes. Int Immunol (1994) 6:1393–402. 10.1093/intimm/6.9.1393 7819148

[93] Zuklys S Balciunaite G Agarwal A Fasler-Kan E Palmer E Hollander GA. Normal thymic architecture and negative selection are associated with Aire expression, the gene defective in the autoimmune-polyendocrinopathy-candidiasis-ectodermal dystrophy (APECED). J Immunol (2000) 165:1976–83. 10.4049/jimmunol.165.4.1976 10925280

[94] Akiyama T Shimo Y Yanai H Qin J Ohshima D Maruyama Y The tumor necrosis factor family receptors RANK and CD40 cooperatively establish the thymic medullary microenvironment and self-tolerance. Immunity (2008) 29:423–37. 10.1016/j.immuni.2008.06.015 18799149

[95] Mouri Y Yano M Shinzawa M Shimo Y Hirota F Nishikawa Y Lymphotoxin signal promotes thymic organogenesis by eliciting RANK expression in the embryonic thymic stroma. J Immunol (2011) 186(9):5047–57. 10.4049/jimmunol.1003533 21441458

[96] Irla M Guerri L Guenot J Serge A Lantz O Liston A Antigen recognition by autoreactive cd4(+) thymocytes drives homeostasis of the thymic medulla. PLoS One (2012) 7:e52591. 10.1371/journal.pone.0052591 23300712 PMC3531460

[97] Roberts NA White AJ Jenkinson WE Turchinovich G Nakamura K Withers DR Rank signaling links the development of invariant gammadelta T cell progenitors and Aire(+) medullary epithelium. Immunity (2012) 36:427–37. 10.1016/j.immuni.2012.01.016 22425250 PMC3368267

[98] White AJ Withers DR Parnell SM Scott HS Finke D Lane PJ Sequential phases in the development of Aire-expressing medullary thymic epithelial cells involve distinct cellular input. Eur J Immunol (2008) 38:942–7. 10.1002/eji.200738052 18350550

[99] Hikosaka Y Nitta T Ohigashi I Yano K Ishimaru N Hayashi Y The cytokine RANKL produced by positively selected thymocytes fosters medullary thymic epithelial cells that express autoimmune regulator. Immunity (2008) 29:438–50. 10.1016/j.immuni.2008.06.018 18799150

[100] Irla M Hugues S Gill J Nitta T Hikosaka Y Williams IR Autoantigen-specific interactions with CD4+ thymocytes control mature medullary thymic epithelial cell cellularity. Immunity (2008) 29:451–63. 10.1016/j.immuni.2008.08.007 18799151

[101] Desanti GE Cowan JE Baik S Parnell SM White AJ Penninger JM Developmentally regulated availability of RANKL and CD40 ligand reveals distinct mechanisms of fetal and adult cross-talk in the thymus medulla. J Immunol (2012) 189:5519–26. 10.4049/jimmunol.1201815 23152561 PMC3605790

[102] Irla M Hollander G Reith W. Control of central self-tolerance induction by autoreactive CD4+ thymocytes. Trends Immunol (2010) 31:71–9. 10.1016/j.it.2009.11.002 20004147

[103] White AJ Jenkinson WE Cowan JE Parnell SM Bacon A Jones ND An essential role for medullary thymic epithelial cells during the intrathymic development of invariant NKT cells. J Immunol (2014) 192:2659–66. 10.4049/jimmunol.1303057 24510964 PMC3948113

[104] Anderson M Anderson SK Farr AG. Thymic vasculature: organizer of the medullary epithelial compartment? Int Immunol (2000) 12:1105–10. 10.1093/intimm/12.7.1105 10882422

[105] Irla M Guenot J Sealy G Reith W Imhof BA Serge A. Three-dimensional visualization of the mouse thymus organization in health and immunodeficiency. J Immunol (2013) 190:586–96. 10.4049/jimmunol.1200119 23248258

[106] Sergé A Bailly AL Aurrand-Lions M Imhof BA Irla M. For3D: full organ reconstruction in 3D, an automatized tool for deciphering the complexity of lymphoid organs. J Immunol Methods (2015). 10.1016/j.jim.2015.04.019 25956038

[107] Rodewald HR Paul S Haller C Bluethmann H Blum C. Thymus medulla consisting of epithelial islets each derived from a single progenitor. Nature (2001) 414:763–8. 10.1038/414763a 11742403

[108] Livet J Weissman TA Kang H Draft RW Lu J Bennis RA Transgenic strategies for combinatorial expression of fluorescent proteins in the nervous system. Nature (2007) 450:56–62. 10.1038/nature06293 17972876

[109] Lind EF Prockop SE Porritt HE Petrie HT. Mapping precursor movement through the postnatal thymus reveals specific microenvironments supporting defined stages of early lymphoid development. J Exp Med (2001) 194:127–34. 10.1084/jem.194.2.127 11457887 PMC2193450

[110] Takahama Y. Journey through the thymus: stromal guides for T-cell development and selection. Nat Rev Immunol (2006) 6:127–35. 10.1038/nri1781 16491137

[111] Petrie HT Zuniga-Pflucker JC. Zoned out: functional mapping of stromal signaling microenvironments in the thymus. Annu Rev Immunol (2007) 25:649–79. 10.1146/annurev.immunol.23.021704.115715 17291187

[112] Guerau-de-Arellano M Martinic M Benoist C Mathis D. Neonatal tolerance revisited: a perinatal window for Aire control of autoimmunity. J Exp Med (2009) 206:1245–52. 10.1084/jem.20090300 19487417 PMC2715060

[113] Ueno T Saito F Gray DH Kuse S Hieshima K Nakano H CCR7 signals are essential for cortex-medulla migration of developing thymocytes. J Exp Med (2004) 200:493–505. 10.1084/jem.20040643 15302902 PMC2211934

[114] Nitta T Nitta S Lei Y Lipp M Takahama Y. CCR7-mediated migration of developing thymocytes to the medulla is essential for negative selection to tissue-restricted antigens. Proc Natl Acad Sci U S A (2009) 106:17129–33. 10.1073/pnas.0906956106 19805112 PMC2761327

[115] Nasreen M Ueno T Saito F Takahama Y. In vivo treatment of class II MHC-deficient mice with anti-TCR antibody restores the generation of circulating CD4 T cells and optimal architecture of thymic medulla. J Immunol (2003) 171:3394–400. 10.4049/jimmunol.171.7.3394 14500633

[116] Williams JA Zhang J Jeon H Nitta T Ohigashi I Klug D Thymic medullary epithelium and thymocyte self-tolerance require cooperation between CD28-CD80/86 and CD40-CD40L costimulatory pathways. J Immunol (2014) 192:630–40. 10.4049/jimmunol.1302550 24337745 PMC3897934

[117] Boehm T Scheu S Pfeffer K Bleul CC. Thymic medullary epithelial cell differentiation, thymocyte emigration, and the control of autoimmunity require lympho-epithelial cross talk via LTbetaR. J Exp Med (2003) 198:757–69. 10.1084/jem.20030794 12953095 PMC2194183

[118] Venanzi ES Gray DH Benoist C Mathis D. Lymphotoxin pathway and Aire influences on thymic medullary epithelial cells are unconnected. J Immunol (2007) 179:5693–700. 10.4049/jimmunol.179.9.5693 17947641

[119] White AJ Nakamura K Jenkinson WE Saini M Sinclair C Seddon B Lymphotoxin signals from positively selected thymocytes regulate the terminal differentiation of medullary thymic epithelial cells. J Immunol (2010) 185:4769–76. 10.4049/jimmunol.1002151 20861360 PMC3826119

[120] Zhu M Chin RK Tumanov AV Liu X Fu YX. Lymphotoxin beta receptor is required for the migration and selection of autoreactive T cells in thymic medulla. J Immunol (2007) 179:8069–75. 10.4049/jimmunol.179.12.8069 18056347

[121] Seach N Ueno T Fletcher AL Lowen T Mattesich M Engwerda CR The lymphotoxin pathway regulates Aire-independent expression of ectopic genes and chemokines in thymic stromal cells. J Immunol (2008) 180:5384–92. 10.4049/jimmunol.180.8.5384 18390720 PMC2760078

